# Primary Osseous Low-Grade Myxofibrosarcoma of Clavicle Presenting With Multiple Skeletal Metastases

**DOI:** 10.7759/cureus.10170

**Published:** 2020-08-31

**Authors:** Kanika Goel, Alan Slipak, Lisa Ercolano, Jan F Silverman, Bang T Tang

**Affiliations:** 1 Department of Pathology, Allegheny Health Network, Pittsburgh, USA; 2 Department of Orthopedics, Allegheny Health Network, Pittsburgh, USA; 3 Department of Pathology, University of North Carolina, Chapel Hill, USA

**Keywords:** myxofibrosarcoma, osseous, skeletal metastases

## Abstract

Myxofibrosarcoma is a soft tissue neoplasm that usually affects the extremities of the elderly. It usually presents as a slow-growing painless mass that can metastasize to the lung and bone. However, the reported incidence of primary osseous myxofibrosarcoma is very low. Moreover, the metastatic pattern of these bone tumors is largely unknown. We describe a unique case of a young Caucasian male with symptomatic low-grade myxofibrosarcoma arising in the left clavicle, who presented with multiple bone metastases.

## Introduction

Myxofibrosarcoma, previously known as “myxoid variant of malignant fibrous histiocytoma”, is a spindle cell neoplasm with a wide spectrum of nuclear atypia and cellularity, embedded in a variably myxoid stroma with typical curvilinear blood vessels [1]. Although direct bone invasion and bone metastases are a known phenomenon related to soft tissue myxofibrosarcoma, there are very few reported cases of a primary osseous origin [2-7]. Moreover, our extensive literature search did not reveal any reported case of bone to bone metastasis with regards to this tumor. We hereby describe this unique case of primary low-grade myxofibrosarcoma arising in the left clavicle, which upon work-up was found to have multiple skeletal metastases.

## Case presentation

**Figure 1:**
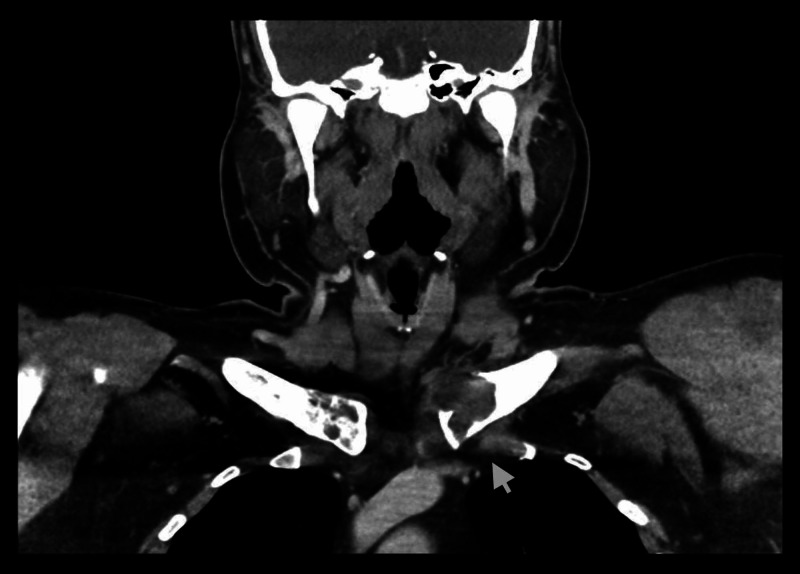
CT scan demonstrating a lytic lesion in left clavicular head

**Figure 2:**
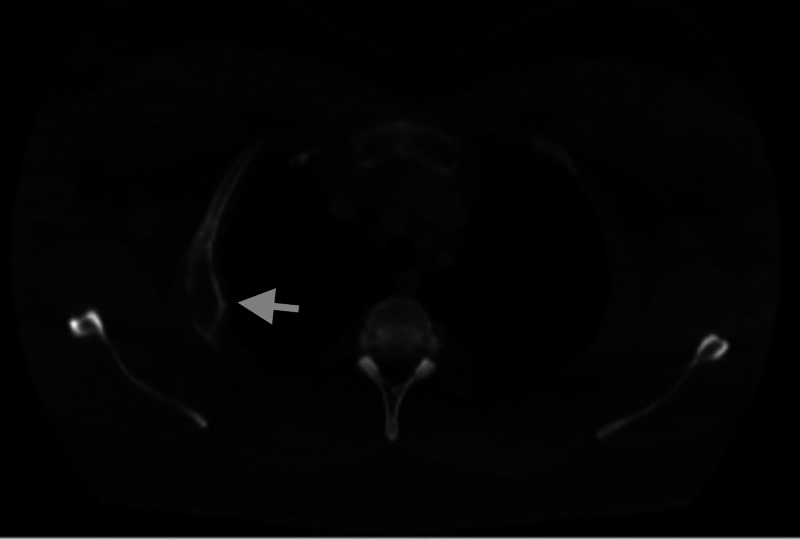
CT scan demonstrating expansile lytic lesion in the second right rib

A 32-year-old Caucasian male with a history of diabetes mellitus noted mild discomfort and swelling of the left shoulder in November 2016. Despite analgesics and six weeks of physical therapy, his pain continued to worsen. Due to persistent symptoms, a CT of the neck was obtained that showed a 3.3-cm expansile lytic lesion at the left clavicular head (Figure 1). An additional expansile lytic lesion was present in the second right rib (Figure 2).

Biopsy of the clavicle lesion performed in March 2017 demonstrated a low-grade spindle cell neoplasm composed of cytologically bland, relatively uniform, spindle cells arranged in a storiform pattern in a fibrous and myxoid stroma. Immunohistochemical (IHC) stain for MUC-4, which is a sensitive and specific marker for low-grade fibromyxoid sarcoma, appeared negative in the scant tissue available. Differential diagnosis at this time included a low-grade fibrosarcoma, desmoplastic fibroma and possibly a low-grade fibromyxoid sarcoma.

**Figure 3:**
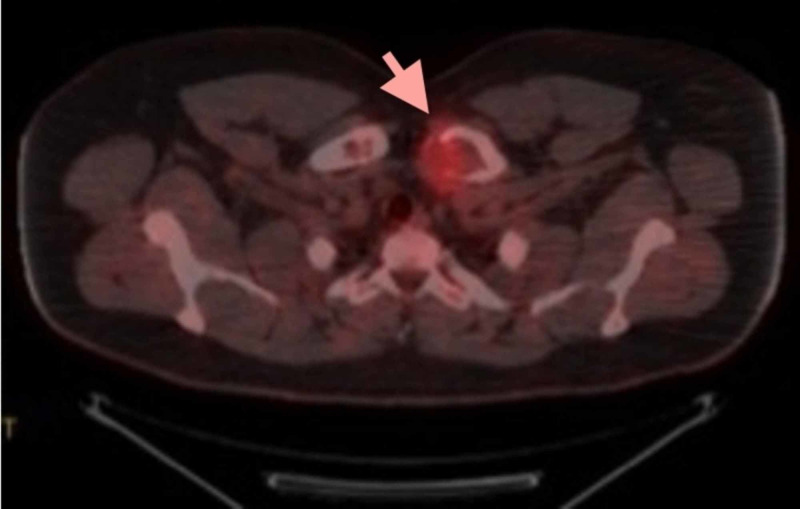
Positron emission tomography (PET) scan demonstrating F-18 fluorodeoxyglucose (FDG)-avid lytic lesion in the left clavicle

**Figure 4:**
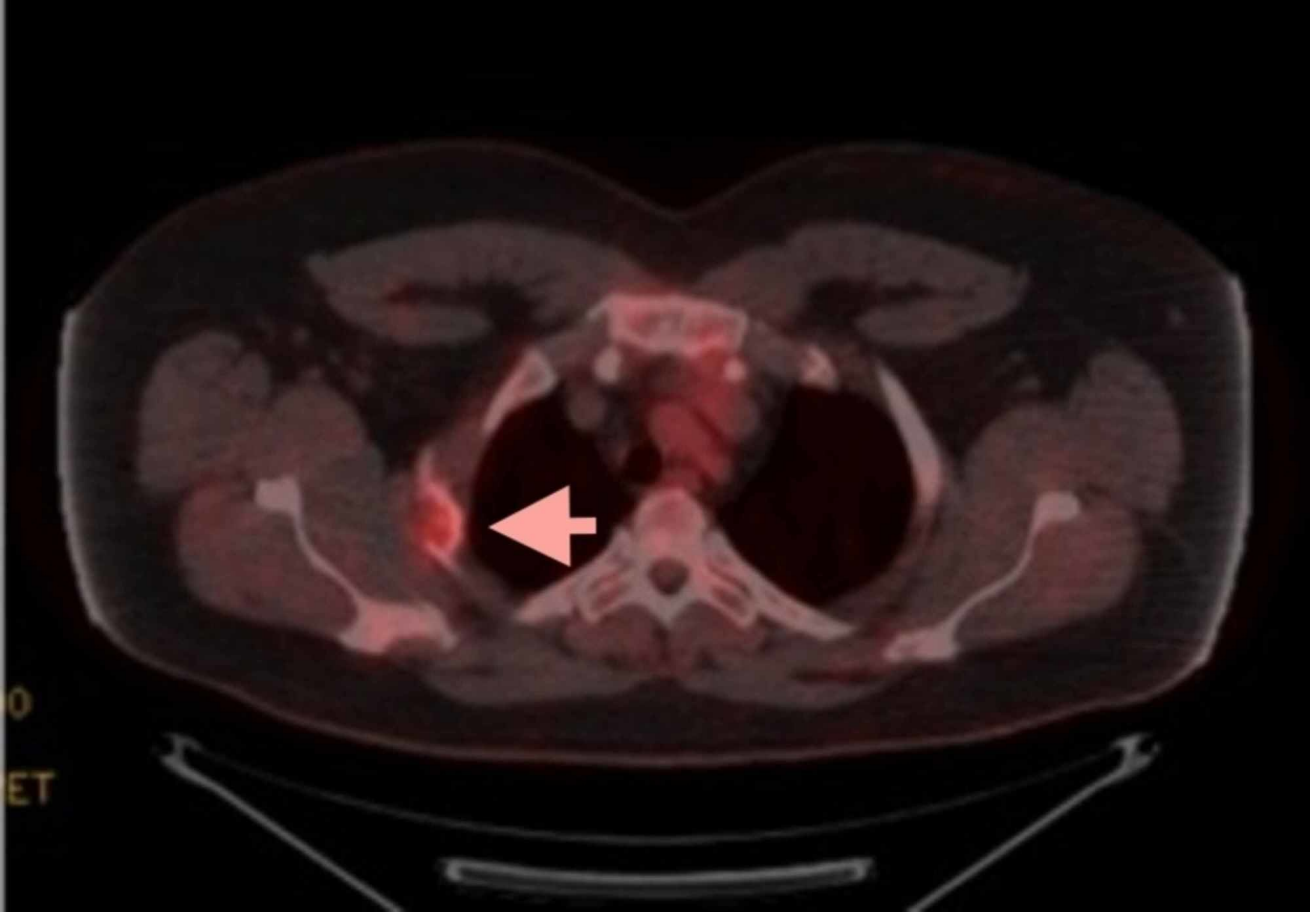
Positron emission tomography (PET) scan demonstrating F-18 fluorodeoxyglucose (FDG)-avid lytic lesion in the second right rib

A positron emission tomography CT (PET-CT) scan was then performed, which demonstrated hypermetabolic (F-18 fluorodeoxyglucose [FDG]-avid), lytic lesions involving the left clavicular head (4.1 × 2.5 cm; Figure 3), second right rib (Figure 4) and proximal left humerus. Lytic lesions were also present in the right ilium and sacrum, but these demonstrated little to no FDG uptake. No soft tissue mass was identified.

**Figure 5:**
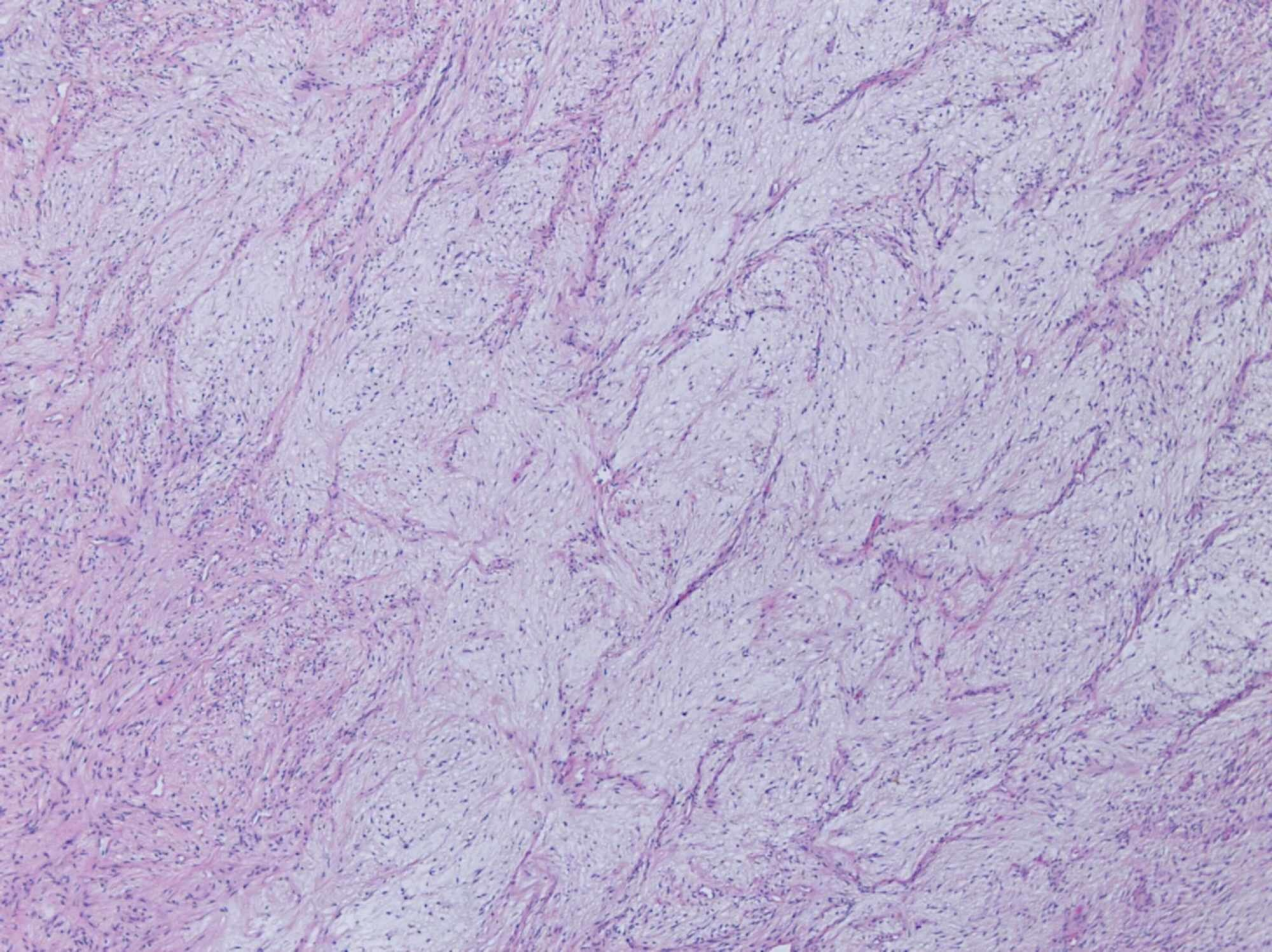
Open biopsy of the left clavicular head (100x magnification): At low power, the biopsy shows low-grade myxofibrosarcoma, grade 1/3 characterized by a spindle cell neoplasm in a fibromyxoid stroma with prominent, arching, interconnecting, curvilinear blood vessels with a perivascular condensation of tumor cells

**Figure 6:**
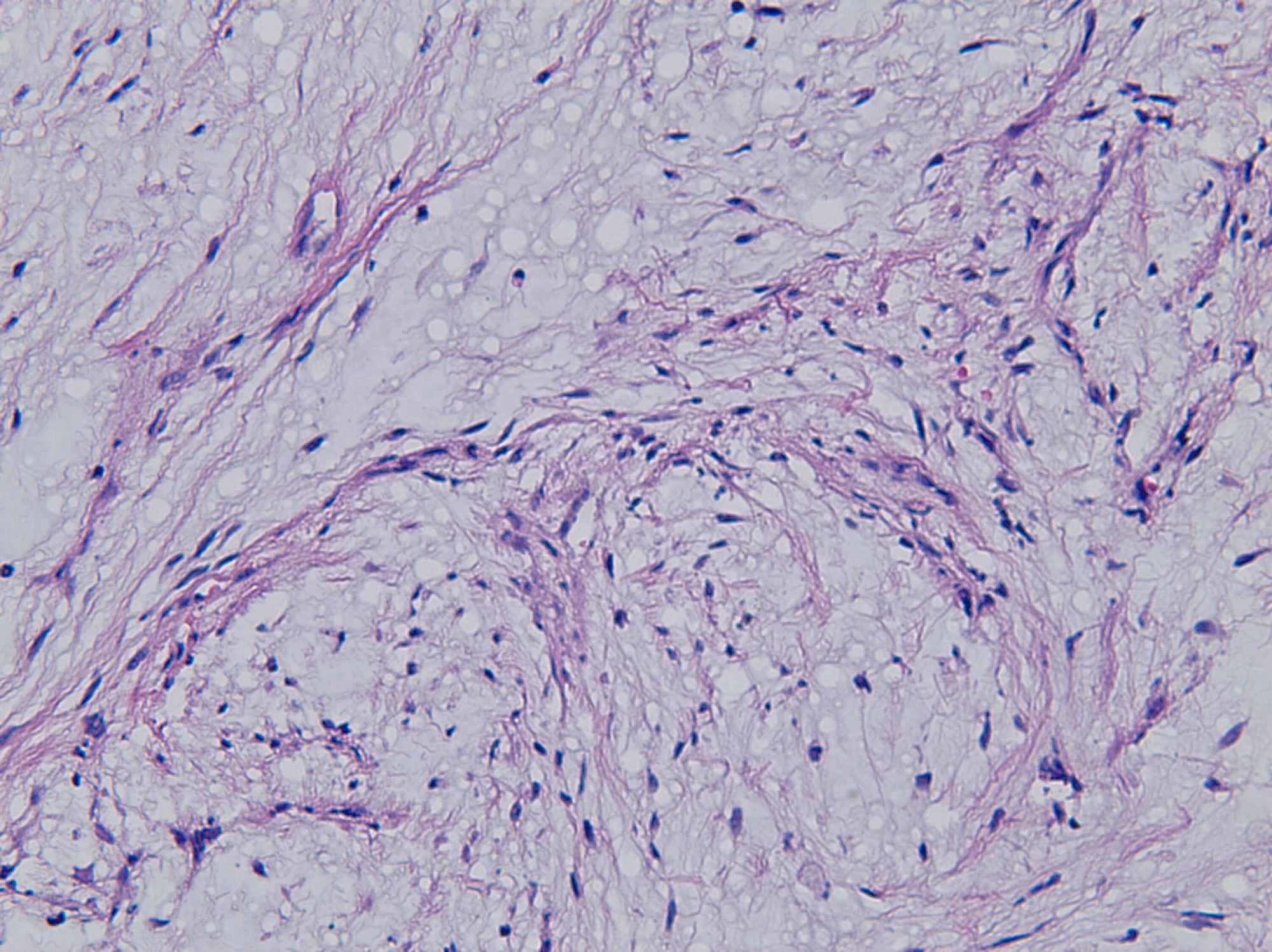
Open biopsy of the left clavicular head (400x magnification): At high power, spindle cells demonstrate indistinct cell margins, slightly eosinophilic cytoplasm and atypical hyperchromatic nuclei

A subsequent CT-guided core biopsy of the right iliac lesion performed in April 2017 showed spindle cell proliferation and hypercellular bone marrow elements. IHC staining for cytokeratin AE1/AE3, desmin, smooth muscle actin (SMA), S100, MUC4 and CD117 was negative with a patchy positivity for CD34. These non-diagnostic needle biopsies prompted an open biopsy of the left clavicular head in May 2017 (Figures 5, 6), which showed spindle and stellate cells enmeshed in a variably myxocollagenous stroma with up to four mitoses per 10 high power fields (HPF). Area of focal necrosis consistent with prior biopsy site changes was also noted. There were also prominent elongated, thin-walled blood vessels and occasional vacuolated cells with cytoplasmic mucin (pseudolipoblasts). On IHC staining, the tumor was focally positive for EMA and CD34 and was negative for keratin, SMA, desmin, MUC4 and S100. Ki-67 proliferation index was 5%-10%. The patient was diagnosed with low-grade (grade 1) myxofibrosarcoma. An open biopsy of the right posterior iliac lesion was also undertaken to determine multifocal versus metastatic disease. This biopsy also showed a spindle cell sarcoma that had similar morphologic features as the non-myxoid component of the clavicular myxofibrosarcoma. The IHC stains for MUC4, ALK1 and MDM2 were negative. This lesion represented a metastatic focus of myxofibrosarcoma. The patient was staged as metastatic primary myxofibrosarcoma of the clavicle with multiple bone metastases, AJCC stage pT1NxM1.

Due to the presence of multiple bone lesions, a potentially curative surgical resection to remove all the lesions was not feasible. In October 2017, he underwent palliative resection of the left clavicular mass with left proximal humerus lesion curettage, cementation and plating. Repeat CT scans demonstrated progressive disease with a marked increase in tumor burden with new skeletal lesions, involving the sacrum, lumbar and thoracic spine, iliac bones, sternum, right clavicle and scapula. Due to the widespread nature of the disease, radiation therapy was not a feasible treatment option. In November 2017, he was started on systemic chemotherapy consisting of cisplatin plus adriamycin alternated with methotrexate (MAP regimen) for metastatic primary myxofibrosarcoma of the bone. However, he developed acute kidney injury and elevation of liver transaminases after cycle 1, requiring interruption of chemotherapy. He would then go on to require prophylactic nailing of the left femur for impending fracture in April 2018. In June 2018, he was found to have new pulmonary metastases, and he eventually succumbed to his progressive disease in December 2018. 

## Discussion

Myxofibrosarcoma is a soft tissue neoplasm accounting for approximately 20% of all soft tissue sarcomas [8]. In 1977, Weiss and Enzinger introduced the term “myxoid variant of malignant fibrous histiocytoma (MFH)” to describe this entity [9]. However, in the absence of a true “histiocytic” origin, the use of this nomenclature seemed unjustified [10]. The term “myxofibrosarcoma” was, therefore, proposed by Angervall et al. [11], which highlighted the myxoid matrix and implied a fibroblastic origin of the tumor [10]. In subsequent years, this tumor came to be increasingly recognized as a distinct neoplasm of fibroblastic origin without histiocytic differentiation [5,10]. The myxoid MFH category was consequently dropped by the World Health Organization (WHO) in the 2002 classification, and myxofibrosarcoma was added to the category of fibrous tumors [5]. WHO defines myxofibrosarcoma as a “spectrum of malignant fibroblastic neoplasms with variably prominent myxoid stroma, cellular pleomorphism and a distinctive curvilinear vascular pattern” [1]. Myxofibrosarcoma was initially graded in a four-tiered system [12]. However, to demonstrate a continuity between low- and high-grade lesions, in 1996, Mentzel introduced a three-tiered grading of the neoplasm into low-, intermediate-, and high-grade depending on the degree of cytologic atypia and the presence or absence of pleomorphic MFH-like lesion within the tumor [10].

Myxofibrosarcoma shows a wide spectrum of cellularity, atypia and proliferative activity; however, distinctive histomorphological features such as multinodular growth with incomplete fibrous septa and a myxoid stroma are present in all cases [1,10]. This neoplasm is characterized by the presence of typical elongated, thin-walled curvilinear vessels associated with a perivascular condensation of tumor cells and/or inflammatory cells [2,4]. Pseudolipoblasts are frequently seen on histological sections [2]. The tumor cells are fusiform, round or stellate with indistinct cell margins, slightly eosinophilic cytoplasm and atypical hyperchromatic nuclei [10]. They exhibit non-specific staining pattern with vimentin and occasional focal staining with SMA and CD34 [1,2,10]. In the absence of a specific diagnostic immunomarker, the diagnosis of myxofibrosarcoma is essentially based on histology. The primary role of immunohistochemical stains in the diagnosis of this entity is to help eliminate other differential diagnoses such as a tumor of lipomatous or neural origin [2]. Establishing a specific molecular profile for diagnosis of this tumor has not been possible so far [2,4].

Myxofibrosarcoma is one of the most common sarcomas of the elderly, usually presenting as a slow-growing painless mass on the extremities [1,10,12]. Other sites of involvement include trunk, head and neck, retroperitoneum, pelvis and heart [1,13]. Approximately 50% of the lesions occur in the dermal or subcutaneous tissue, while the remainder occur in the underlying fascia and skeletal muscle [1,10,12]. Invasion of the underlying bone by primary tumor has also been rarely reported [13-15]. Primary treatment is surgical resection for limited disease, but local recurrence is common as is seen in almost half of the cases [1,10]. Although there is no known impact of the grade of tumor on rate of local recurrence, a higher grade is associated with an increased risk of metastases and mortality [2,4].

Myxofibrosarcoma is known to metastasize to the bone [1], but the incidence of primary osseous myxofibrosarcoma (myxofibrosarcoma arising in the bone) is extremely rare, with only a handful of cases reported in the literature. The first published case of primary osseous myxofibrosarcoma was by Frassica et al. in 1988, with the tumor arising in the left distal femur of a 49-year-old male [4]. It was called myxoid fibrosarcoma at that time. Two cases of myxoid malignant fibrous histiocytoma of the skull were described in 1997 and 2002 [7,16]. In 2004, a low-grade myxofibrosarcoma of tibia was reported by Kapur and Sarode [6]. In 2010, Zhang et al. described myxofibrosarcoma involving the frontal bone at the lateral superior aspect of the orbit in a 27-year-old female [5]. In 2012, Romeo et al. re-evaluated 67 cases of previously diagnosed (between 1990 and 2009) "malignant fibrous histiocytoma" or "fibrosarcoma" of bone, and three of these cases were reclassified as myxofibrosarcoma of bone involving the lower extremities [3]. In 2017, Kayser et al. reported myxofibrosarcoma in an area of osteonecrosis of distal femur [2].

## Conclusions

Myxofibrosarcoma is a myxoid soft tissue neoplasm, with an extremely rare incidence of a primary osseous origin. To the best of our knowledge, this is the first reported case of primary myxofibrosarcoma of the bone presenting with multiple skeletal metastases (bone to bone metastases).

Due to the use of a variable nomenclature in relation to the tumor in the past, the true incidence of this entity may have been underestimated. The absence of a specific immunostaining pattern with no known diagnostic molecular markers may have further contributed to the low reported incidence of this neoplasm.

## References

[1] Fletcher CD, Bridge JA, Hogendoorn PC, Mertens F (2013). WHO Classification of Tumors of Soft Tissue and Bone, 4th Edition. https://www.ncbi.nlm.nih.gov/nlmcatalog/101622555.

[2] Kayser D, Walton Z, Bruner E, Chapin RW (2017). Myxofibrosarcoma: first report of myxofibrosarcoma of bone arising at a bone infarct. Skeletal Radiol.

[3] Romeo S, Bovee JV, Kroon HM (2012). Malignant fibrous histiocytoma and fibrosarcoma of bone: a re-assessment in the light of currently employed morphological, immunohistochemical and molecular approaches. Virchows Arch.

[4] Frassica FJ, Sim EH, Wold LE (1988). Case report 462: grade 2 myxoid fibrosarcoma of femur. Skeletal Radiol.

[5] Zhang Q, Wojno TH, Yaffe BM, Grossniklaus HE (2010). Myxofibrosarcoma of the orbit: a clinicopathologic case report. Ophthalmic Plast Reconstr Surg.

[6] Kapur P, Sarode V (2004). Pathologic quiz case: myxoid tibial lesion in a 31-year-old man. Low-grade myxofibrosarcoma. Arch Pathol Lab Med.

[7] Nakayama K, Nemoto Y, Inoue Y (1997). Malignant fibrous histiocytoma of the temporal bone with endocranial extension. AJNR Am J Neuroradiol.

[8] Tsuchie H, Kaya M, Nagasawa H (2017). Distant metastasis in patients with myxofibrosarcoma. Ups J Med Sci.

[9] Weiss SW, Enzinger FM (1977). Myxoid variant of malignant fibrous histiocytoma. Cancer.

[10] Mentzel T, Calonje E, Wadden C, Camplejohn RS, Beham A, Smith MA, Fletcher CD (1996). Myxofibrosarcoma. Clinicopathologic analysis of 75 cases with emphasis on the low-grade variant. Am J Surg Pathol.

[11] Angervall L, Kindblom LG, Merck C (1977). Myxofibrosarcoma. A study of 30 cases. Acta Pathol Microbiol Scand A.

[12] Merck C, Angervall L, Kindblom LG, Oden A (1983). Myxofibrosarcoma. A malignant soft tissue tumor of fibroblastic-histiocytic origin. A clinicopathologic and prognostic study of 110 cases using multivariate analysis. Acta Pathol Microbiol Immunol Scand Suppl.

[13] Li W, Li D, Zhu X, Lu S, He C, Yang Q (2014). Low-grade myxofibrosarcoma following a metal implantation in femur: a case report. Diagn Pathol.

[14] Park SW, Kim HJ, Lee JH, Ko YH (2009). Malignant fibrous histiocytoma of the head and neck: CT and MR imaging findings. AJNR Am J Neuroradiol.

[15] Murphey MD, Gross TM, Rosenthal HG (1994). From the archives of the AFIP. Musculoskeletal malignant fibrous histiocytoma: radiologic-pathologic correlation. Radiographics.

[16] Woodhams R, Kan S, Iwabuchi K, Oka H, Sagiuchi T, Hayakawa K (2002). A case of malignant fibrous histiocytoma (MFH) in the skull bone presenting extensive permeative osteolysis. Radiat Med.

